# Association between Nfr2, HO-1, NF-kB Expression, Plasma ADMA, and Oxidative Stress in Metabolic Syndrome

**DOI:** 10.3390/ijms242317067

**Published:** 2023-12-02

**Authors:** Ganka Y. Bekyarova, Deyana G. Vankova, Valentina H. Madjova, Nicolai A. Bekyarov, Ayshe S. Salim, Diana G. Ivanova, Stefka M. Stoeva, Daniela I. Gerova, Yoana D. Kiselova-Kaneva

**Affiliations:** 1 Department of Physiology and Pathophysiology, Medical University of Varna, 9002 Varna, Bulgaria; 2 Department of Biochemistry, Molecular Medicine and Nutrigenomics, Medical University of Varna, 9002 Varna, Bulgariaayshe.salim@mu-varna.bg (A.S.S.); divanova@mu-varna.bg (D.G.I.); stefka.stoeva@mu-varna.bg (S.M.S.); 3 Department of General Medicine, Medical University of Varna, 9002 Varna, Bulgaria; valentina.madjova@mu-varna.bg (V.H.M.);; 4 Department of Clinical Laboratory, Medical University Varna, 9002 Varna, Bulgaria

**Keywords:** Nrf2, NF-kB, HO-1, PBMC, plasma ADMA, oxidative stress, endothelial dysfunction

## Abstract

Endothelial dysfunction is one of the major factors in the pathogenesis of metabolic syndrome (MetS), and its molecular mechanisms are not completely understood. The present study aimed to examine the connection between nuclear factor2-related factor2 (Nrf2), nuclear factor kappa-light-chain-enhancer of activated B cells (NF-κB), heme oxygenase 1 (HO-1), and plasma asymmetric dimethylarginine (ADMA) and malondialdehyde (MDA) in people with MetS. Participants in the study were as follows: with MetS (*n* = 30) and without MetS (Control) (*n* = 14). Expression of Nrf2, NF-kB, and HO-1 was measured in peripheral blood mononuclear cells (PBMCs). Plasma ADMA was determined using the ELISA technique and MDA via the thiobarbituric acid method. Our study showed that mRNA of NF-kB, Nrf2, and HO-1 levels in PBMCs in the MetS group were significantly higher than in the controls by 53%, 130%, and 185% (*p* < 0.05), respectively. Similarly, elevated levels of MDA (by 78%, *p* < 0.001) and ADMA (by 18.7%, *p* < 0.001) were established in the MetS group. Our findings show the importance of transcription factor Nrf2, playing an integral role in the protection of the endothelium, and of NF-κB, a transcription factor mediating the inflammatory response in MetS. Knowledge of complex cellular–molecular mechanisms would allow the use of biomarkers such as Nrf2, NF-kB, HO-1, and ADMA for the assessment of endothelial dysfunction in clinical practice.

## 1. Introduction

Endothelial dysfunction (ED) is characterized by decreased bioavailability of nitric oxide (NO) and impaired endothelial-dependent vasodilation, one of the major risk factors of metabolic syndrome (MetS) [1,2]. Endothelial dysfunction is considered to be a key factor in the development of vascular diseases [3]. Asymmetric dimethylarginine (ADMA) is an endogenous competitive inhibitor of nitric oxide synthase [4,5]. Increased ADMA has been also linked to the presence of hypertension, type 2 diabetes, and obesity as components of MetS [6,7,8,9]. The ED’s molecular mechanisms in MetS are not completely understood, but free radicals and inflammatory mediators are thought to be involved. Recent evidence suggests that oxidative stress and inflammation induced by high low density lipoproteins (LDL) or by TNF-α (Tumor necrosis factor alpha) increase ADMA levels, and, respectively, decrease NO bioavailability [6]. Reactive oxygen species (ROS) enhance NO degradation and uncouple endothelial nitric oxide synthase (eNOS) to redirect the enzyme to generate ROS [10]. 

Heme oxygenase 1 (HO-1) is an enzyme induced by nuclear factor2-related factor2 (Nrf2) and plays a critical role in antioxidant defense in response to various inflammatory and oxidative stimuli [1]. HO-1 expression significantly suppresses the production of proinflammatory mediators and reverses impaired eNOS expression in response to oxidised LDL and TNF-α [11]. 

Although HO-1 is expressed at low levels in endothelial cells and white blood cells under physiological conditions, it is highly inducible in response to various pathophysiological stresses/stimuli [12,13]. Heme-oxygenase-1 catalyzes the degradation of heme to carbon monoxide and bilirubin, which also have cytoprotective effects for endothelial dysfunction against various stresses [12,14,15]. The beneficial effect of HO-1 expression in the attenuation of metabolic syndrome, obesity, cardiovascular disease, and diabetic cardiomyopathy has been reported [16,17]. 

Nrf2, a redox-sensitive transcription factor, transcribes many antioxidant genes. Nfr2 is important in the transcriptional activation of HO-1 and several other genes that contain specific sequences called Antioxidant Response Elements (AREs) [18]. There is insufficient data on the role of HO-1 in the pathogenesis of endothelial dysfunction in MetS. It has been reported that HO-1 protects endothelial cells against oxidative stress in aging, hypertension, diabetes, and atherosclerosis [19,20]. Nrf2 decreases oxidative stress by regulating the expression of antioxidant genes while ADMA enhances oxidative stress by inhibiting the nitric oxide pathway via competitive inhibition of the nitric oxide synthase pathway [21]. Nrf2/ARE signaling has recently been shown to play an important role in endothelial protection under oxidative stress [22]. 

Inflammation plays an important role in ED in MetS and the inhibition of endothelial nitric oxide synthesis by ADMA and may be related to increased concentrations of inflammatory mediators [23]. Previous studies demonstrated that plasma ADMA could concentration-dependently increase ROS production, and that these ROS consequently act as second messengers and stimulate the activation of uclear factor kappa-light-chain-enhancer of activated B cells (NF-kB) in endothelial cells [24]. NF-κB is a key transcription factor in the expression of proinflammatory markers such as TNF-α, intercellular adhesion molecule 1 (ICAM-1), and vascular cell adhesion protein 1 (VCAM-1) released by white blood cells [25,26]. Data on investigations of Nrf2, NF-KB transcription factors in humans, and their role in the pathogenesis of ED in MetS are very scarce. 

The present study aimed to examine the connection between Nrf2 as a critical regulator of defense against oxidative stress, NF-κB, a transcription factor mediating the inflammatory response, HO-1, endothelial protector and antioxidant, plasma ADMA, and MDA in people with MetS. 

## 2. Results

The basic demographic characteristics of the participants are presented in Table 1.

Thirty subjects (83.5%) met NCEP-ATPIII criteria for metabolic syndrome diagnosis. These subjects showed higher levels of WC, BMI, TG, HDL, and systolic and diastolic pressures.

**Figure 1:**
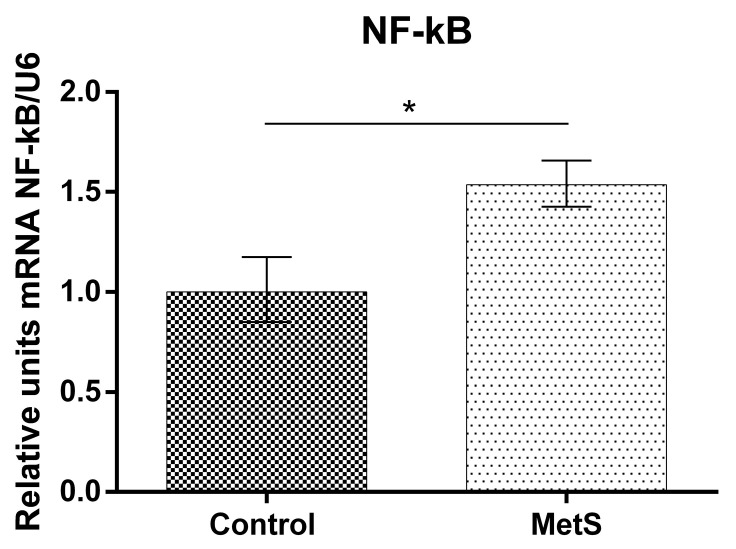
Nuclear factor kappa-light-chain-enhancer of activated B cells (NF-kB) gene expression in Peripheral Blood Mononuclear Cells (PBMCs) of subjects with metabolic syndrome (MetS) and control subjects (Control). Data are presented as mean ± SEM (statistical significance was indicated at *p* < 0.05), * *p* < 0.05.

**Figure 2:**
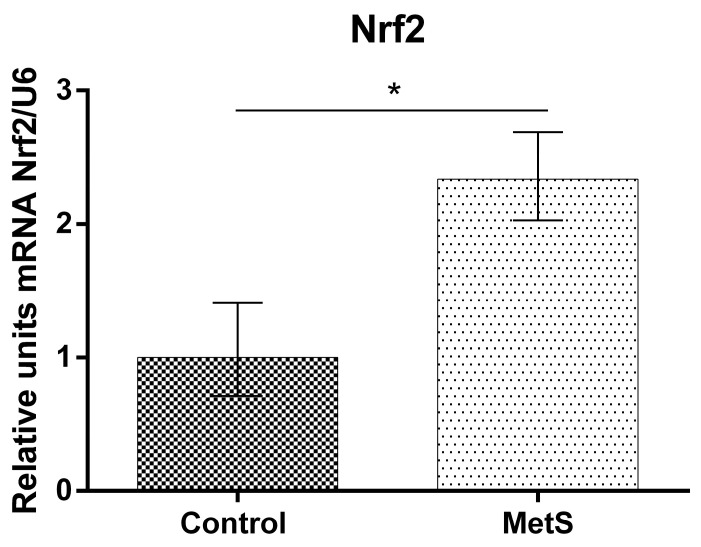
Nuclear factor2-related factor2 (Nrf2) gene expression in PBMCs of subjects with MetS and control subjects (Control). Data are presented as mean ± SEM (statistical significance was indicated at *p* < 0.05), * *p* < 0.05.

**Figure 3:**
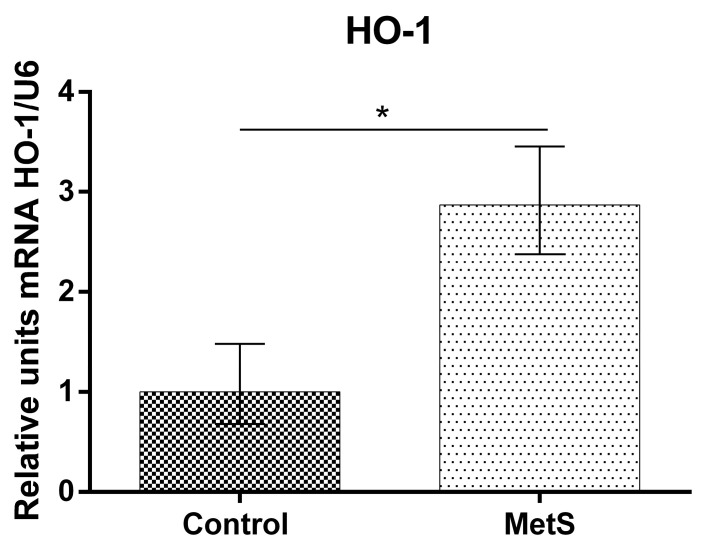
Heme oxygenase 1 (HO-1) gene expression in PBMCs of subjects with MetS and control subjects (Control). Data are presented as mean ± SEM (statistical significance was indicated at *p* < 0.05) *, *p* < 0.05.

Our study showed that NF-KB mRNA (over 53%), Nrf2 mRNA (over 130%), and HO-1 mRNA expression (over 185%) in peripheral blood mononuclear cells (PBMCs) in the MetS group were elevated compared to control subjects (Figure 1, Figure 2 and Figure 3).

**Figure 4:**
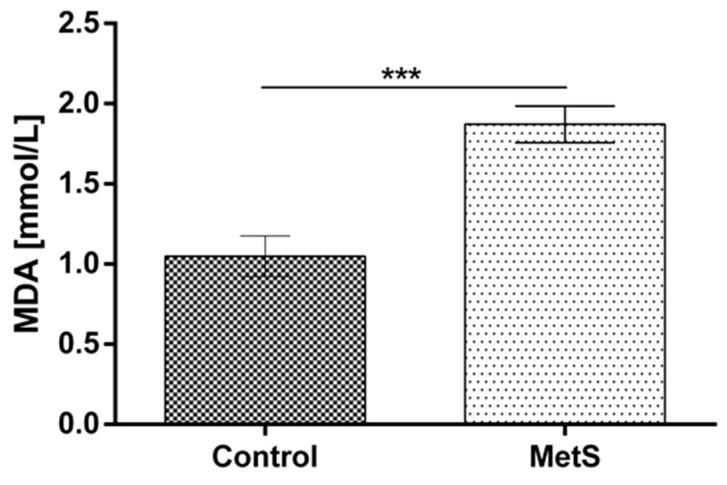
Plasma malondialdehyde (MDA) levels in subjects with MetS and control subjects (Control). Data are presented as mean ± SEM (statistical significance was indicated at *p* < 0.05), *** *p* < 0.001.

**Figure 5:**
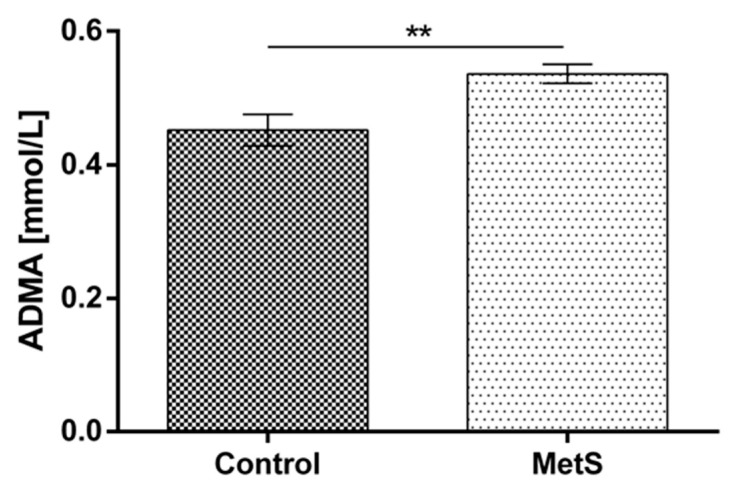
Plasma asymmetric dimethylarginine (ADMA) levels in subjects with MetS and control subjects (Control). Data are presented as mean ± SEM (statistical significance was indicated at *p* < 0.05), ** *p* < 0.01.

Plasma MDA was increased significantly in the metabolic group vs. the control by 78% (1.05 ± 0.13 to 1.87 ± 0.11) (Figure 4). Plasma ADMA was also increased by 18.7% (0.45 ± 0.02 to 0.54 ± 0.01) (Figure 5).

**Figure 6:**
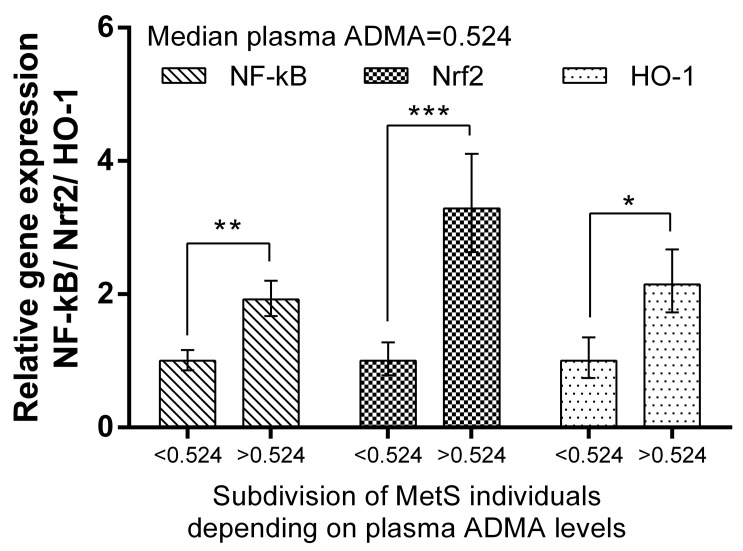
Differential expression of NF-kB, Nrf2, and HO-1 in MetS subgroups, depending on plasma ADMA levels. Subdivision was as follows: subgroup with ADMA plasma levels ≤ 0.524 mmol/L (median value). Data are presented as mean ± SEM (statistical significance was indicated at *p* < 0.05), * *p* < 0.05; ** *p* < 0.01; *** *p* < 0.001.

**Figure 7:**
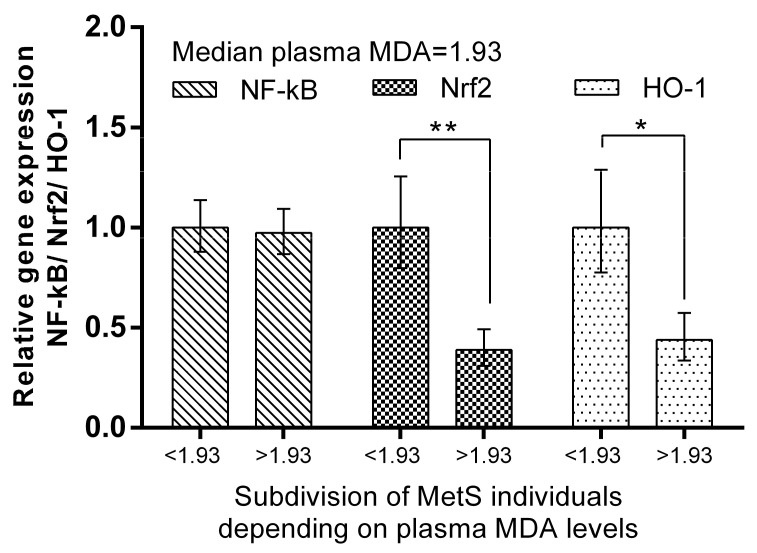
Differential expression of NF-kB, Nrf2, and HO-1 in MetS subgroups, depending on plasma MDA levels. Subdivision was as follows: subgroup with MDA plasma levels ≤ 1.93 mmol/L (median value). Data are presented as mean ± SEM (statistical significance was indicated at *p* < 0.05), * *p* < 0.05; ** *p* < 0.01.

We subdivided MetS individuals into two groups according their plasma ADMA levels: ADMA ≤ median (=0.524 mmol/L) and ADMA > median of the group. The expression levels of Nrf2 and HO-1 in PBMCs were more than 320% (*p* < 0.001) and 214% (*p* < 0.01), respectively, higher in the MetS subgroup with ADMA > median. Significantly higher levels (192%, *p* < 0.01) of NF-kB mRNA were also detected (Figure 6). The same subgrouping according to plasma MDA levels revealed a lower relative expression of Nrf2 (by 61%, *p* < 0.01) and HO-1 (by 56%, *p* < 0.05) in the subgroup with plasma MDA higher than the median (=1.930 mmol/L). NF-kB was not found to be differently expressed, depending on plasma MDA levels in the MetS group (Figure 7). 

## 3. Discussion

In the present study, we investigated the expression of NF-κB, a transcription factor mediating the inflammatory response, Nrf2 as a critical regulator of defense against oxidative stress, HO-1, an endothelial protector and antioxidant, and the relationship between them and plasma MDA and ADMA in people with MetS. 

ADMA, as a marker of endothelial dysfunction, and MDA, as a marker of oxidative stress and lipid peroxidation, are increased in MetS. Some studies report a significant association between increased ADMA levels, hypercholesterolemia, and endothelial dysfunction in people with MetS [1,27,28]. It is well known that hypercholesterolemia and increased ADMA and MDA levels lead to endothelial dysfunction, which is considered a key factor in the development of proatherogenic changes in endothelial cell and vascular events, including stroke and myocardial infarction. Hypercholesterolemia may disturb the function or regulation of dimethylarginine dimethylaminohydrolase (DDAH), thereby leading to intracellular accumulation of ADMA and increased oxidative stress [29].

In our study, we found a positive association between plasma MDA, a marker of oxidative stress, and plasma ADMA, a marker of endothelial dysfunction in individuals with MetS. It is known that ADMA is metabolized by DDAH to dimethylamine and citrulline [30,31]. Oxidative stress, TNF-α, homocysteine, and nitrosative stress have been shown to reduce DDAH activity. Increased plasma concentrations of ADMA were found to cause an increase in superoxide (O_2_^−^) production by nicotinamide adenine dinucleotide phosphate (NADPH) oxidase, thereby leading to oxidative stress and decreased NO bioavailability [32]. ROS and products of lipid peroxidation are crucial factors for the activation of transcriptional factors Nrf2 and NF-kB [33,34]. As nuclear factor Nrf2r is located in the cytoplasm, its translocation to the nucleus is triggered by different stress stimuli which have the potential to disturb cellular redox balance [18,35,36]. Therefore, in stressful milieu such as oxidative/inflammatory insults, Nrf2 moves to the nucleus and binds to its ARE sites to cause the upregulation of enzymes to counteract the effect of increased ROS.

**Figure 8:**
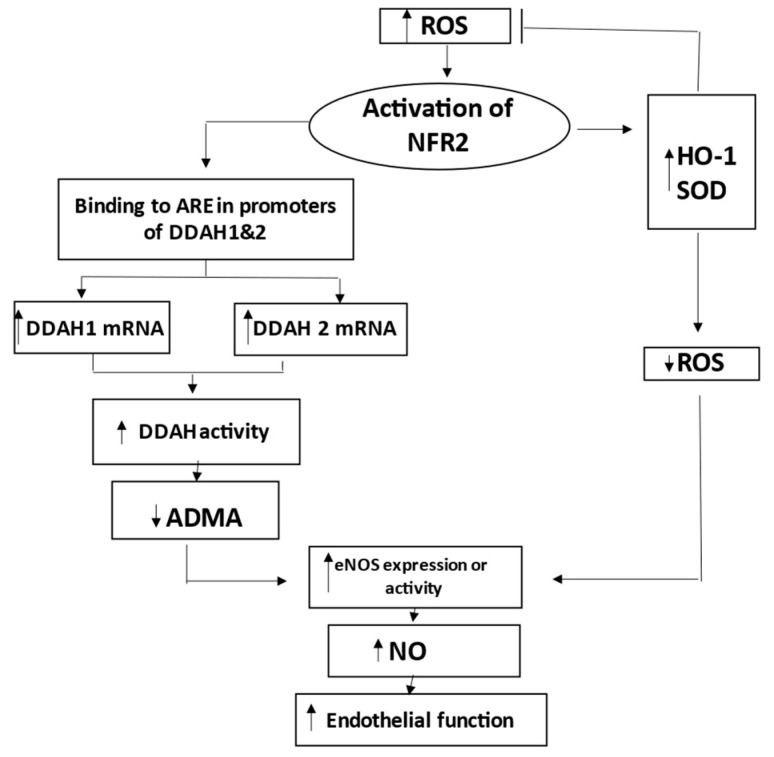
Schematic representation of the role of nuclear factor erythroid 2-related factor 2 (Nrf2) in preserving endothelial function during activation by reactive oxygen species (ROS). Nrf2 increases expression of HO-1 and other genes to decrease ROS. Nrf2 also increases DDAH-1 and DDAH-2, leading to degradation and decrease of ADMA, which is inhibitor of eNOS. Decreased levels of ADMA result in improved NO production and related endothelial function. Thus, Nrf2 coordinates HO-1/DDAH/eNOS pathways that enhance endothelial NO generation and endothelial function. ADMA indicates asymmetric dimethylarginine; ARE, antioxidant response element; DDAH, dimethylarginine dimethylaminohydrolase; eNOS, endothelial nitric oxide synthase; HO-1, hemoxygenase-1; and SOD, superoxide dismutase.

Nrf2 may activate DDAH-1, which metabolizes ADMA, the endogenous inhibitor of nitric oxide synthase eNOS [31]. Increased DDAH-1 is accompanied by decreased levels of ADMA in endothelium and plasma. This would support endothelial function through adequate NO production and the homeostasis of endothelial function [21]. Therefore, Nrf2 can enhance endothelial NO generation and protect endothelial function by regulating the DDAH/ADMA/eNOS pathway (Figure 8). 

HO-1 is an Nrf2-dependent gene that exerts beneficial effects through protection against oxidative damage [15]. HO-1 is important in attenuating the overall production of reactive oxygen species (ROS) through its ability to degrade heme and produce carbon monoxide and biliverdin/bilirubin, and release free iron [37]. In clinical cases, human HO-1 deficiency causes oxidative stress and endothelial cell injury [14]. In contrast, the up-regulation of HO-1 protein may represent an attempt to minimize cellular injury [38]. Biliverdin and CO are gaining particular interest because these two have been found to mediate most of the anti-inflammatory, antioxidant, and anti-apoptotic effects of HO-1. The present study showed increased HO-1 expression in PBMCs of MetS subjects. Elevated plasma HO-1 levels have been reported in diabetes [39] as well as in other chronic diseases [40,41,42]. HO-1 is an Nrf2-dependent gene that exerts beneficial effects through endothelial protection against oxidative damage [37]. 

The activation of Nrf2 promoting HO-1 expression can inhibit oxidative stress and increase eNOS activity and NO production [43]. Furthermore, HO-1 induction inhibits ROS production in the aorta isolated from animals with fructose-induced MetS [44]. HO-1, by modulating the activation of Nrf2, sets an adaptive reprogramming that enhances antioxidant defenses [45]. It has been reported that the increased HO-1 expression and heme degradation products can improve vascular function, at least in part, by compensating for the loss of NO bioavailability [43].

Nrf2 increases the expression of HO-1 and other genes to decrease ROS, as well as increases DDAH-1 and DDAH-2 to decrease ADMA and to increase the eNOS-mediated bioavailability of NO [31]. Therefore, Nrf2 coordinates HO-1/DDAH/eNOS pathways that enhance endothelial NO generation and endothelial function (Figure 8). 

Previous studies demonstrated that ADMA could concentration-dependently increase the production of ROS, which act as second messengers and activate NF-kB in endothelial cells [46]. The data from the present study showed an increased NF-κB expression in PBMCs, which corresponds to higher plasma MDA and ADMA levels in MetS. ADMA may activate NF-κB, the expression of cytokines, soluble ICAM-1 and VCAM-1 expression, and monocyte adhesiveness, and may mediate their proinflammatory actions in the endothelium [23]. It has been proven that the reduced eNOS/NO activity activates NF-κB responsible for the expression of endothelin-1 and other proinflammatory mediators including TNF-α, interleukin 6 (IL-6), free radicals, chemokines, and adhesives molecules (ICAM-1, endothelial leucocyte adhesion molecule-1 (ELAM-1)), as well as inducible NO synthase (iNOS) contributing to endothelial dysfunction. 

ADMA is produced in many tissues and is known to induce the expression of Nrf2, which in response stimulates DDAH [47]. In this study, we subdivided MetS individuals in two groups according to their plasma ADMA levels: ADMA ≤ median (=0.524 mmol/L) and ADMA > median of the group. Expression levels of Nrf2 in PBMCs were more than 320% (*p* < 0.0.001) higher in the MetS subgroup with ADMA > median. Similarly, HO-1, which is a target of Nrf2, was, correspondingly, 214% (*p* < 0.01) higher in the MetS subgroup with high ADMA plasma levels. Significantly higher levels (192%, *p* < 0.01) of mRNA were also detected for NF-kB in the same subgroup (Figure 6). Data suggest that the gene expression of respective genes in PBMCs may reflect plasma ADMA levels in metabolic syndrome. In support of this hypothesis is the observation that, under ADMA treatment, cells, including endothelial, produce ROS and upregulate NF-kB [25,48]. 

The same subgrouping according to plasma MDA levels revealed lower relative expression of Nrf2 (by 61%, *p* < 0.01) and HO-1 (by 56%, *p* < 0.05) in the subgroup with plasma MDA higher than the median (=1.930 mmol/L). At the same time, NF-kB, which is related to inflammation pathways [49], was not found to be differently expressed depending on plasma MDA levels in the MetS group (Figure 7). This observation suggests other mechanisms by which the expression of respective genes in PBMCs is dependent on MDA plasma levels in metabolic syndrome. In pathological conditions, cells may have decreased HO-1 expression. For example, this enzyme is downregulated in PBMCs in multiple sclerosis [50]. Further studies would reveal whether impaired balance of the protective mechanisms in PBMCs in MetS could be a reason for this observation. 

## 4. Materials and Methods

### 4.1. Participants 

This study was approved by the local Ethical Committee at the Medical University of Varna (Protocol № 86/26.09.2019). Written informed consent was obtained from all participants in the study. A total of 44 people (39 females, 5 males) were included in the study. The participants of the study were divided into two groups: those newly diagnosed with metabolic syndrome (MetS) (n = 30), and nonmetabolic, clinically healthy individuals (Control) (n = 14). Individuals in the MetS group were selected according to CEP-ATP III criteria [51] (the presence of three or more of the following: waist circumference ≥102 cm (men), ≥88 cm (women); blood pressure ≥130/85 mm Hg or treatment for hypertension; triglycerides ≥ 1.7 mmol/L (≥150 mg/dL); HDL-cholesterol < 1.03 mmol/L (<40 mg/dL) for men, <1.29 mmol/L (<50 mg/dL) for women). The participants were newly diagnosed and indicated as MetS based on the abovementioned criteria and were not under treatment for metabolic syndrome.

For biochemical analyses, venous fasting blood was collected in vacutainers (EDTA). Plasma was separated by centrifugation at 1500 rpm for 15 min, aliquoted, and stored at −20 °C. Subsequent analyses were performed immediately after the thawing of the samples. Plasma lipid parameters (total cholesterol, LDL-cholesterol, HDL-cholesterol, and triglycerides) were measured using routine methods. 

### 4.2. ADMA and MDA Measurement

Plasma ADMA concentrations were determined using the ELISA assay kit (DLD Diagnostica GMBH, Germany) after it was validated locally. The method was validated in compliance with the international standard of quality and competence of medical laboratories (BDS/EN/ISO 15189). Measurements were performed in duplicate. Data are presented in mM/L. 

Lipid peroxidation was assayed via MDA, measured according to its thiobarbituric acid (TBA) reactivity in plasma using the method of Porter et al. [52]. Data are presented in mmol/L. 

### 4.3. Peripheral Blood Mononuclear Cells (PBMCs) Collection 

Whole blood samples were used for peripheral blood mononuclear cells collection. Blood (6 mL) was collected in lithium heparin vacutainer tubes. The separation of PBMCs was carried out using a Ficoll separation medium with a density of 1.077 g/L and 50 mL LeucoSep™ centrifuge separation tubes (GreinerBioOne, Kremsmünster, Austria) containing a porous barrier, which enables cell separation by means of density gradient centrifugation and purification following the manufacturer’s instructions. 

### 4.4. Gene Expression Analysis 

RNA was extracted from collected PBMCs with Tri reagent (Ambion^®^, Life Technologies, Waltham, USA) using the manufacturer’s protocol. First-strand cDNA synthesis was performed with 0.1 µg of total RNA using a RevertAid First Strand cDNA Synthesis Kit (ThermoScientific, Waltham, MA, USA),a nd using (dT)18 primer and following the steps in the manufacturer’s instructions. Quantitative gene expression analysis was performed using two-step real-time qPCR with SYBR Green qPCR 1 × Master Mix with ROX (KAPA SYBR FAST qPCR Kit, KAPA BIOSYSTEMS, USA). The primer sequences used were as follows: U6 Forward GCTTCGGCAGCACATATACTAAAAT; Reverse CGCTTCACGAATTTGCGTGTCAT; HMOX1 Forward TCAGGCAGAGGGTGATAGAAG; Reverse TTGGTGTCATGGGTCAGC; NRF2 Forward TCCAGTCAGAAACCAGTGGAT; Reverse GAATGTCTGCGCCAAAAGCTG; NF-kB Forward AGAGGCTTCCGATTTCGATATGG; Reverse GGATAGGTCTTTCGGCCCTTC. Analysis was performed on an Applied Biosystems^®^ 7500 Real-Time qPCR instrument (Waltham, USA). The amount of mRNA of each gene of interest was normalized according to the amount of mRNA encoding U6 as an internal control. Gene expression levels were calculated using the 2^−ΔΔCt^ method. Gene expression levels of respective genes were presented in relative units as compared to the control group, where expression was considered to be equal to 1 as described by Livak and Schmittgen [53]. 

### 4.5. Statistical Analysis 

Data were presented as mean ± SEM or percentage (%), as appropriate. Data analysis was performed on GraphPad Prism v. 8.3. and SPSS v. 23. Standard statistical methods such as descriptive statistics, unpaired Student’s *t*-tests for normally distributed parameters, and one-way ANOVA with Bonferroni correction were used.

## 5. Conclusions

In conclusion, our findings show the importance of transcription factor Nrf2, playing an integral role in the protection of the endothelium and of NF-κB, the transcription factor mediating the inflammatory response in in MetS. Investigating the relationship between MDA, ADMA, HO-1, and the transcription factors Nrf2 and NF-kB would contribute to elucidating the complex pathophysiological mechanisms of cellular damage in MetS, and this is important for the prevention, early diagnosis, and treatment strategy of cardiovascular diseases and MetS. The knowledge of complex cellular–molecular mechanisms in PBMCs would allow the use of biomarkers such as HO-1, ADMA, Nrf2, and NF- kB for the assessment of imbalance in PBMCs in MetS. 

## Figures and Tables

**Table 1:** Baseline characteristics of the study groups.

Groups	Control *n* = 30	MetS *n* = 14	*p* Value	*p* Value Adjusted by Age
Age	40 ± 1.11	44 ± 1.27	0.001	
BMI kg/m	22.99 ± 0.51	29.13 ± 0.69	0.0001	0.001
WC	74.60 ± 2.53	93.00 ± 1.3	0.0001	0.001
SBP mmHg	123.00 ± 3.01	144.00 ± 1.07	0.0001	0.001
DBP mmHg	79.50 ± 1.89	90.10 ± 0.99	0.0001	0.001
TG mmol/L	0.70 ± 0.07	1.72 ± 0.12	0.0001	0.001
HDL mmol/L	2.28 ± 0.27	1.38 ± 0.07	0.0001	0.001

Data expressed as mean ± SEM. MetS indicates metabolic syndrome; BMI, body mass index; WC, waist circumference; SBP, systolic blood pressure; DBP, diastolic blood pressure; HDL, high-density lipoprotein; TG, triglycerides.

## Data Availability

The data presented in this study are available upon reasonable request.
